# The role of resveratrol on rheumatoid arthritis: From bench to bedside

**DOI:** 10.3389/fphar.2022.829677

**Published:** 2022-08-22

**Authors:** Shuyan Sheng, Xinyi Wang, Xin Liu, Xinyang Hu, Yubao Shao, Gaoyuan Wang, Deshen Mao, Conghan Li, Bangjie Chen, Xiaoyu Chen

**Affiliations:** ^1^ First Clinical Medical College of Anhui Medical University, Hefei, China; ^2^ School of pharmacy, Anhui Medical University, Hefei, China; ^3^ School of Basic Medical Sciences, Anhui Medical University, Hefei, China; ^4^ Microscopic Morphological Center Laboratory, Anhui Medical University, Hefei, China; ^5^ First Affiliated Hospital of Anhui Medical University, Hefei, China; ^6^ Anhui Provincial Laboratory of Inflammatory and Immune Diseases, Hefei, China

**Keywords:** rheumatoid arthritis, resveratrol, SIRT1, NF-κB, drug delivery system

## Abstract

Rheumatoid arthritis (RA) is a systemic autoimmune disease characterized by symmetrical polyarthritis as its main clinical manifestation. Uncontrolled RA eventually leads to joint deformities and loss of function. Currently, the pathogenesis of RA remains under discussion, and RA treatment is still at the bottleneck stage. Resveratrol has long been regarded as a potential antioxidant drug for RA treatment. Currently, resveratrol is considered to exert therapeutic effects on RA by activating silent information regulator 1 (SIRT1) and its downstream pathways. There is notable crosstalk between the SIRT1 and NF-κB pathways, and these pathways, which play an essential role in the development of RA, are unexpectedly linked to the influence of resveratrol. Based on recent studies of almost all the pathways that resveratrol can affect, this review summarizes a regulatory chain of core components that cover multiple tracks. We also list the effects of resveratrol on immune cells and other subtle controls, which can help clinicians understand the known mechanism of resveratrol and better treat patients with RA.

## Introduction

Rheumatoid arthritis (RA) is a chronic autoimmune disease of unknown etiology. The basic pathological changes include synovitis and pannus formation, progressive bone destruction, joint deformity, and loss of joint function (Harris, 1990; Yanni et al., 1994; McInnes and Schett, 2011). In addition, an abnormal production of autoantibodies, especially rheumatoid factor (RF) and anti-citrullinated protein/peptide antibodies (ACPA), is usually regarded as a vital component of the disease (Gavrilă et al., 2016). The disease is most common in women (Crowson et al., 2011) and the elderly, where 50 percent of the risk can be attributed to genetic factors, with smoking being the main environmental factor (Scott et al., 2010). Methotrexate (MTX) is a frontline drug for RA (Singh et al., 2016); however, a third of patients do not show an adequate response to MTX due to insufficient improvement of the disease symptoms or the appearance of side effects that result in treatment discontinuation (Klareskog et al., 2004). Thus, the biological agents represented by tumor necrosis factors (TNFs) has become a reasonable choice for second-line treatment. When necessary, joint replacement may be performed. The current goal of RA treatment is to minimize joint pain and swelling and prevent visible deformities to allow patients t0 work normally and participate in personal activities to improve their quality of life (Wasserman, 2011).

Resveratrol (3,5,4′-trihydroxy trans-stilbene) is a natural polyphenol found in grapes, red wine, grape juice, and several kinds of berries (Nunes et al., 2018) and is considered an antioxidant, anti-inflammatory, anti-apoptotic, and anti-cancer drug (Shakibaei et al., 2009; Hsieh and Wu, 2010; Kalantari and Das, 2010; Chan et al., 2016; Blanquer-Rosselló et al., 2017; Jardim et al., 2018). It has been discovered in different areas of the world, and resveratrol varies slightly from place to place (Cock and van Vuuren, 2014; Cock et al., 2015). Resveratrol is a free radical scavenger; however, its direct scavenging activity is relatively poor, and its antioxidant properties *in vivo* are more likely due to its role as a gene regulator. For example, resveratrol inhibits NADPH oxidase-mediated ROS production by downregulating the expression and activity of NADPH oxidase (Xia et al., 2017; Breuss et al., 2019). It is important to note that most of the regulation of these genes by resveratrol is mediated by sirtuin 1 (SIRT1) or Nrf2. SIRT1 plays a critical role in the ability of moderate doses of resveratrol to stimulate AMPK and improve mitochondrial function *in vitro* and *in vivo* (Price et al., 2012). In muscle cells, resveratrol activates AMPK, increases SIRT1 and PGC-1α protein levels, increases citrate synthase activity without altering mitochondrial content, and improves muscle mitochondrial respiration on fatty acid-derived substrates (Timmers et al., 2011). In addition, resveratrol has been shown to protect mitochondria and regulate redox biology and kinetics in *in vitro* and *in vivo* experimental models. (Valero, 2014; Gibellini et al., 2015; Tellone et al., 2015; de Oliveira et al., 2016)

The possible therapeutic effect of resveratrol on RA has attracted the attention of researchers worldwide, and considerable study results have been obtained. At present, many reviews on resveratrol have clarified its anti-cancer (Carter et al., 2014) and anti-cardiovascular disease effects. Some scholars have also systematically summarized the effects of resveratrol on inflammatory bowel disease (a disease with a mechanism similar to RA). However, there has been no specific review of the anti-RA effects of resveratrol. Therefore, this review summarizes current research on the mechanism of resveratrol in the treatment of RA, including its effect on Sirt1-mediated anti-oxidant gene regulation, mitochondrial protection, and other aspects. In addition, the results of *in vivo* studies that provide evidence for the efficacy of resveratrol, as an adjuvant or single treatment for RA, in relieving symptoms and prevent complications are detailed. It is worth noting that based on the above retrospective studies, this review also focuses on novel ideas and prospects for future clinical applications of resveratrol in combination with the latest research results and technologies in the field of life science and medicine.

## Overview of RA and resveratrol

Resveratrol (3,5,4′-trihydroxy trans-stilbene) is a natural edible polyphenol compound of the stilbene family, which mainly exists in various types of plant foods and is synthesized by more than 70 plants. It was first reported by Japanese scholar Takaoka in 1939 (Hasan and Bae, 2017). Resveratrol has shown a wide range of biological activities in various *in vitro* and *in vivo* experiments, including antioxidant, anti-inflammatory, anti-cancer, anti-aging, and nervous system protection effects. Resveratrol is a popular and novel research item, and its application in various diseases has been widely investigated.

Given its antioxidant, anti-inflammatory, and protective roles in the normal redox function of the mitochondria, resveratrol is likely to play a therapeutic role in the pathophysiology of RA. Elmali et al. first reported in 2005 that resveratrol injection effectively alleviated cartilage degradation in an experimental rabbit osteoarthritis model (OA) induced by unilateral anterior cruciate ligament transection (ACLT) (Elmali et al., 2005). In 2007, it was reported that the injection of resveratrol effectively reduced cartilage damage in an experimental arthritis model (RA) induced by lipopolysaccharide (LPS) (Elmali et al., 2007). These studies provide empirical evidence for the application of resveratrol in RA. As for clinical trials, Hani M. Khojah et al. conducted a clinical randomized controlled trial in 100 patients with RA (68 women, 32 men) in 2018, and the results support the addition of resveratrol as an adjuvant to traditional anti-rheumatic drugs (Khojah et al., 2018).

RA is a common autoimmune disease that is characterized by synovial inflammation and hyperplasia (swelling), autoantibody production (ACPA), cartilage and bone destruction (deformity), and systemic features, including cardiovascular, pulmonary, psychological, and skeletal diseases. Compared with systemic lupus erythematosus (SLE), RA is an erosive arthritis that can cause bone destruction. Specifically, the bone destruction occurs due to an autoimmune inflammatory reaction that concentrates on the synovium and can lead to joint deformity. Synovitis occurs due to changes in RA in the joint. During the acute phase, synovial exudation, congestion, and edema are accompanied by neutrophil infiltration. In the chronic stage, the sliding surface becomes hypertrophic and forms fluffy processes called pannus, which protrude into the articular cavity or invade the cartilage and subchondral bone. Outside the joints, this can manifest as vasculitis. Small and medium arteries and veins are also involved. Rheumatoid nodules are a manifestation of vasculitis, and RA onset is slow. The common initial sites are hand facets, such as the proximal interphalangeal joints (PIP) and metacarpophalangeal joints (MCP), and the condition presents as symmetrical, multiple, and peripheral facet arthritis. The pain occurs repeatedly and worsens after rest, and the clinical manifestations of RA vary significantly among individuals. RA occurs in the joints and can manifest as joint stiffness, pain, tenderness, and swelling. In the late stage, joint muscle atrophy, finger deviation to the ulnar side, metacarpophalangeal joint subluxation, and the appearance of “swan neck” and “button patterns” occurs. Some patients with RA may present or later develop disease manifestations in other organs (sometimes without obvious articular involvement), such as interstitial lung disease (ILD), pericarditis, pleural effusion, or bronchiectasis (Sparks, 2019). The most basic pathological processes of RA include the production of inflammatory factors and the proliferation, invasion and migration of fibroid synovial cells. One of the most important inflammatory factors is TNFα. In RA, TNFα exerts pro-inflammatory effects that induce leukocyte and endothelial cell activation, cytokine and chemokine cascades, angiogenesis, and nociceptor activation (Grell et al., 1995). TNFα can also regulate the generation of osteoclasts and inhibit the differentiation of osteoblasts, thereby breaking the balance between osteoclasts and osteoblasts, resulting in damage to bone and joints (Bertolini et al., 1986; Marahleh et al., 2019). In synovial cells extracted from RA patients, it was found that knocking down TNFα could significantly reduce the production of other pro-inflammatory factors, such as IL-1β, IL-6, IL-8, granulo-macrophage colony-stimulating factor (GM-CSF), *etc*., thereby exerting anti-inflammatory and anti-rheumatic effects (Micheau and Tschopp, 2003). More interestingly, another study found that the expression of IL-1β in macrophages in bronchoalveolar lavage fluid of patients with RA-associated interstitial pneumonia (RA-UIP) was significantly up-regulated, suggesting the role of IL-1β in the pulmonary complications of RA (Shin et al., 2019).

Population-based findings suggest that the prevalence of RA ranges between 0.5 and 1.0 percent among adults in developed countries. In these studies, the prevalence rates in women were three times higher than those in men. The condition is most common in women over 65 years of age, which suggests that hormonal factors may play a pathogenic role (Symmons et al., 2002). The prevalence of RA among adults in developed countries ranges from 5 to 50 per 100,000 people and increases with age (Carbonell et al., 2008), (Pedersen et al., 2009). However, there is also epidemiological evidence that the incidence of RA may decline as its onset is delayed (Doran et al., 2002; Kaipiainen-Seppanen and Kautiainen, 2006). RA prevalence varies geographically (Biver et al., 2009), and the disease is relatively rare in developing countries, such as rural West Africa, compared to North America and Northern Europe (Szapłyko et al., 2003). This suggests that the genetic risk and environmental exposure in different populations play important roles in the development of RA.

The pathogenesis of RA is very complex, is not fully understood, and can be induced by multiple factors, such as heredity, infection, and environment. Heritability is estimated to be 60%, which may be caused by gene mutations in the HLA class II family (Dedmon, 2020). Among them, the HLA-DRB1 allele mutation has the strongest genetic association with RA, which may account for at least 30% of the total inheritance of the disease (van der Helm-van Mil et al., 2005). Infection and autoimmune reactions are central to the pathogenesis of RA. Although there no confirmed direct infection factors have been identified to lead to RA, several studies have shown that smoking can significantly increase the risk of RA.

Current views on immune disorders mainly focus on the abnormal initiation of the innate immune response (such as the activation of the monocyte macrophage system) and adaptive immune response (such as the activation of the T cell system), the abnormal expression of cytokines, growth and differentiation factors, and transcription factors, and the abnormal activation of intracellular signaling pathways. RA is generally considered to be a disease that is mediated by Th1 cells, but more recently, investigators have paid increasing attention to the role of Th17 cells, which can produce IL17A, il17f, IL21, IL22, and TNF, and a series of cytokines that promote the pathological process of RA. In addition, many experimental results have confirmed the role of the NF-κB signaling pathway in the pathogenesis of RA (Zhang et al., 2019; Wang et al., 2020). Coincidentally, resveratrol has been shown to reduce the number and function of Th17 cells in draining lymph nodes (DLN) (Xuzhu et al., 2012; Nguyen N. et al., 2015). The compound can also reduce the ROS production by inhibiting the NF-κB signaling pathway. There are many cases in which the pathogenesis of RA has been found to involve the target of resveratrol, which opens the possibility for the use of resveratrol in RA treatment.

## Molecular mechanism of resveratrol in RA

While the molecular mechanism of resveratrol in RA requires further study, the known mechanisms have largely elucidated the effects of resveratrol. We have compiled comprehensive studies of resveratrol, and provide an overview of new research. We also summarize several core pathways that are mediated by resveratrol, including a regulatory chain based on SIRT1. Other effects of resveratrol, including its role in promoting apoptosis and inhibiting immune cell function, have also been detailed.

### A regulation chain based on SIRT1

SIRT1 participates in a wide variety of cellular processes; however, its molecular mechanism remains largely unknown. Wendling et al. (Wendling et al., 2015) found that in patients with RA, Sirt1 protein levels in peripheral blood mononuclear cells were lower than normal.

#### SIRT1 down-regulates MMP expression

Matrix metalloproteinases (MMPs) are a group of enzymes that degrade various protein components in the extracellular matrix (ECM) and destroy the histological barrier of fibroblast-like synoviocytes (FLS). MMPs are secreted by the synovial pannus into the synovial fluid and are then transferred to the ECM, leading to the degradation of articular bones and cartilages (Miller et al., 2009; Marrelli et al., 2011; Bartok et al., 2014; Li et al., 2015). Aggressive expression of MMP3 and MMP13 has been detected in RA-FLS isolated from patients with RA (Fan et al., 2013). Furthermore, the migration and invasion abilities of RA-FLS were significantly inhibited by the downregulation of MMP1 and MMP13 expression (Gao et al., 2015; Jie et al., 2015). Sirt1 inhibits the invasion of RA-FLS by promoting the decomposition of MMPs, thus inhibiting the development of RA (Hao et al., 2017).

#### SIRT1 down-regulates NF-κB (nuclear factor-κB) expression

The NF-κB signaling pathway and SIRT1 enzymes are evolutionarily conserved mechanisms that maintain cellular homeostasis (Kauppinen et al., 2013). SIRT1 can inhibit NF-κB signaling directly or indirectly, In turn, the NF-κB system suppresses SIRT1-mediated function by inhibiting the downstream targets of SIRT1. Wang et al. (Wang et al., 2014) reported that the induction of Sirt1 levels *via* inhibition of nuclear factor-κB (NF-κB) p65 protein expression improves arthritis in CIA rats. This finding suggests that the antagonistic effects of both may be reflected in the pathogenesis of RA. Furthermore, Yeung et al. (Yeung et al., 2004) demonstrated that SIRT1 directly interacts with and deacetylates the RelA/p65 component of the NF-κB complex. SIRT1 can play an anti-inflammatory role by inhibiting the NF-kB signaling pathway and the production of monocyte inflammatory factors TNF-α, IL-1B, IL6, and Ki-67 (Park et al., 2016). Circulating monocytes infiltrate synovial tissues across synovial blood vessels and differentiate into macrophages, which play a crucial role in the pathogenesis of RA (Alivernini et al., 2020). Notably, activated macrophages are the major source of IL-1 and TNF-α in the synovium of patients with RA. Yang and Karsenty (Yang and Karsenty, 2002) reported PU.1 (a hematopoietic transcription factor) is required for macrophage differentiation, and SIRT1 has been shown to suppress PU.1. This means that SIRT1 regulates RA by inhibiting NF-κB during monocyte differentiation. The inhibition of monocyte differentiation further suppresses pro-inflammatory cytokine production and consequently inhibits the severity of collagen-induced arthritis in mice (Park et al., 2013).

The identification of the role of the MAPK signaling pathway in RA has aroused extensive investigations. Bradykinin (BK) is a potent mediator of pain and swelling and initiates the release of cytokines from leukocytes in arthritis (Bhoola et al., 1992). In FLS, BK activates the B2R receptor, which in turn activates PKCμ, and the activated MAPK signaling pathway can promote the expression of AP-1 (specifically c-Jun and FOS) and NF-KB (specifically p65) to produce COX-2 (el-Dahr et al., 1996; Xie et al., 2000). Many experiments have suggested that Cox-2 is an inducible cyclooxygenase that metabolizes arachidonic acid into prostaglandin E2 (PGE2), a key trigger for subsequent inflammation (Sano et al., 1992; Crofford, 1997). Resveratrol has been found to inhibit NF-KB as well as the above mechanism. Therefore, resveratrol can reduce the production of COX-2 and function as a powerful drug against RA (Tsai et al., 2017; Yang et al., 2017; Dudics et al., 2018a).

In FLS, resveratrol can also inhibit NF-kB through the NF-κB/miR-29a-3p/Keap1/Nrf2/ARE/ROS pathways, and NF-κB/miR-23a-3p/cul3/Nrf2/ARE/ROS inhibition reduces the production of ROS. ROS are one of the key factors that activates upstream MAPK signaling. The reduction in ROS indirectly inhibits the activation of the MAPK signaling pathway, thus inhibiting the production of COX-2 at the upstream level.

**FIGURE 1:**
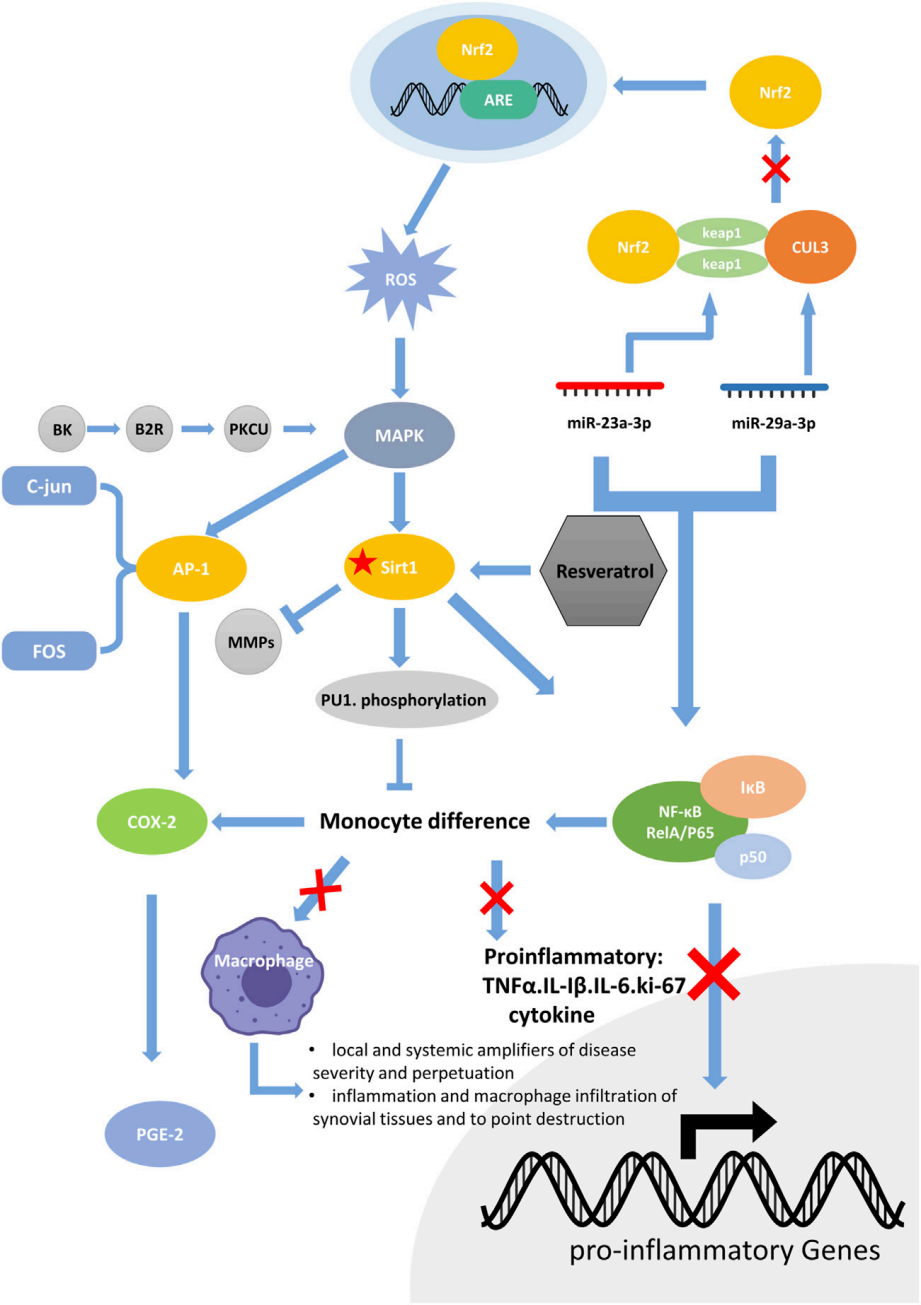
The RA molecular regulation chain surrounding SIRT1. SIRT1 can downregulate NF-κB and MMP1/MMP13, both of which promote the invasion of RA. NF-κB participates in mir-29a-3p/Keap1 and mir-23a-3p/Cul3. They both regulate downstream Nrf2, mediate ARE expression, and produce ROS. On the other hand, BK/B2R/PKCμ Pathway and Nrf2/ARE/ROS pathway can act on downstream MAPK, which can mediate AP-1 and NF-κB, thus forming a closed loop of complex signaling pathways starting from the SIRT1 pathway.

In conclusion, we have summarized the mechanisms and cross-pathways of SIRT1 in RA. SIRT1 can downregulate NF-κB and MMP1/MMP13, both of which promote the invasion of RA. NF-κB participates in mir-29a-3p/Keap1 and mir-23a-3p/Cul3. They both regulate downstream Nrf2, mediate ARE expression, and produce ROS. On the other hand, BK the B2R/PKCμ Pathway and Nrf2/ARE/ROS pathways can act on downstream MAPK, which can mediate AP-1 and NF- κ B, thus forming a closed loop of complex signaling pathways starting from the SIRT1 pathway (Figure 1).

In addition to basic research studies, resveratrol has been widely used in clinical studies to activate SIRT1 to treat RA. Daniel Wendling et al. (Wendling et al., 2014) published a study that highlighted the importance of SIRT1 in RA. They found that SIRT1 activity (nucleus and cytoplasm) was not related to Das28, CRP, ESR, or IL-8, but rather to baseline IL-6 (*p* = 0.02) and TNF (*p* = 0.04) in the RA and control groups. SIRT1 activity in ACPA-positive RA was lower than that in ACPA-negative RA (*p* = 0.01). Furthermore, SIRT1 activity was higher in the corticosteroid group than in the ACPA-negative RA group (*p* = 0.03).

### Pro-apoptotic effects

RSV inhibits the expression of BCL XL in MH7A cells and promotes SIRT1 mediated apoptosis; which enhances the activation of caspase 8 in fls-85 cells, both of which can cause the efficient release of cytochrome c in mitochondria, activate caspase 9 and caspase 3, and promote apoptosis (Byun H. S. et al., 2008; Nakayama et al., 2012b; Nguyen N. T. et al., 2015). In inflammatory cells, an increased level of caspase 3 activation was observed with RSV (Dudics et al., 2018b). Furthermore, a microarray analysis showed that RSV also affects the expression of DNA replication-related genes, p53 pathway genes, PDGF signal pathway genes, ccna2, and rhoj in RA-FLS (Glehr et al., 2013b).

The important pathological features of RA include the proliferation of FLS cells, differentiation and adhesion of macrophages, and infiltration of inflammatory cells. Related experimental results have shown that resveratrol can significantly promote the apoptosis of these cells; therefore, resveratrol has the potential to alleviate and treat RA. The pro-apoptotic function of resveratrol depends mainly on the caspase pathway. However, using the caspase 8 inhibitor z-IETD-fmk in RA-FLS cells can completely eliminate the apoptosis-promoting effect of resveratrol, whereas the caspase 3 inhibitor z-DEVDfmk only partially eliminates the apoptosis-promoting effect of resveratrol. This phenomenon proves that apoptotic pathways mediated by caspase-independent death effect factors (including mitochondrial AIF) may exist (Byun H. et al., 2008).

**FIGURE 2:**
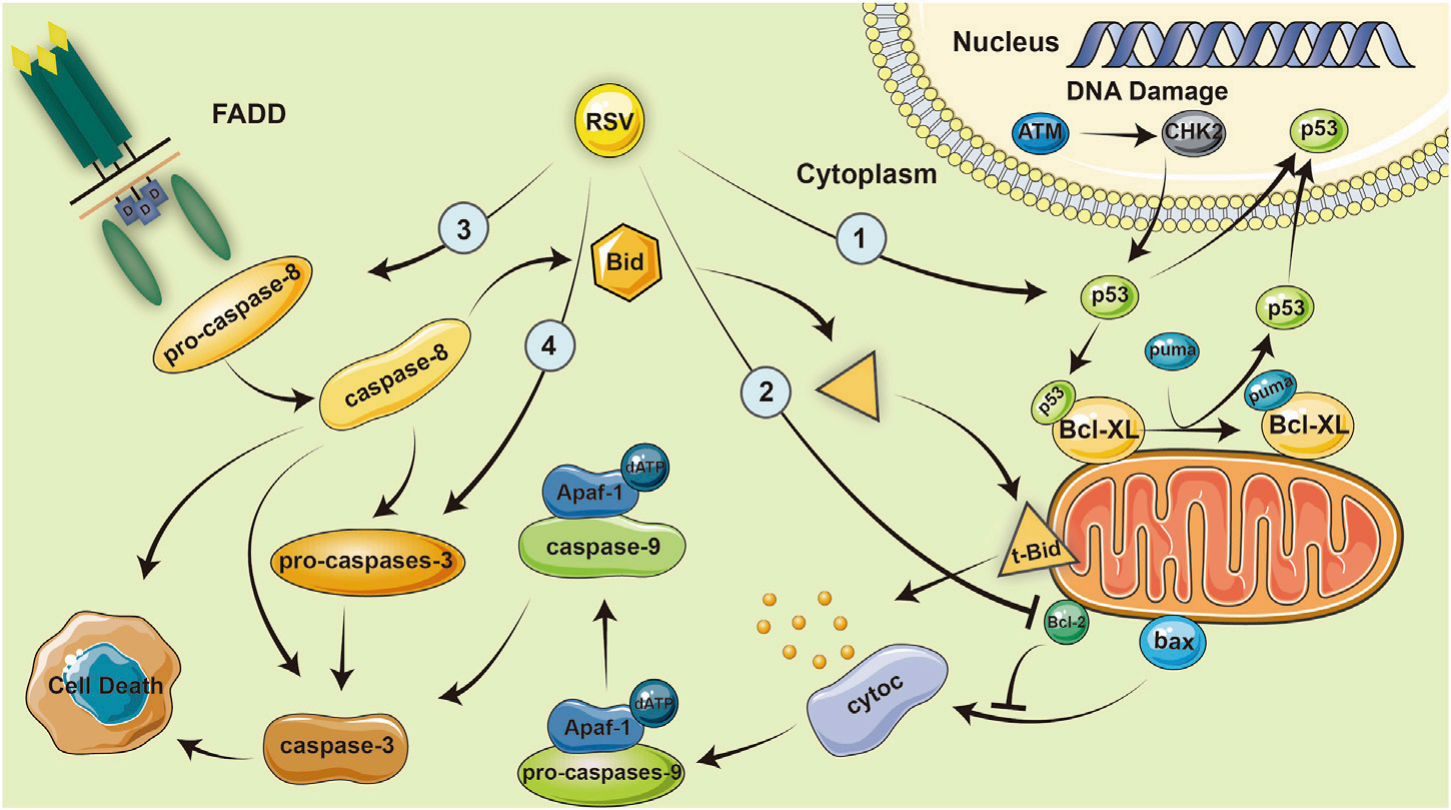
Resveratrol inhibits RA by promoting apoptosis. Resveratrol inhibits the expression of BCL-XL in MH7A cells, which enhances the activation of caspase-8 in fls-85 cells and causes the efficient release of cytoc in mitochondria, eventually activating caspase-9 and caspase-3, and promoting apoptosis.

Using the figure below, we summarize several ways in which resveratrol can inhibit RA by promoting apoptosis (Nakayama et al., 2012a; Glehr et al., 2013a; Nguyen N. et al., 2015; Dudics et al., 2018a) (Figure 2).

### Suppression of immune cell function

The differentiation of mononuclear cells into macrophages plays a central role in the pathophysiology of RA. Macrophages are the primary cellular responder to rheumatoid factor and simulate the secretion of various inflammatory mediators in synovial tissue, which has been considered an important clinical target. Pu.1 is an important member of the transcription factor ETS family and a major regulator of hematopoietic stem cell proliferation and differentiation. Pu.1 regulates immune cell proliferation and differentiation, and plays an important role in the regulation of innate and acquired immunity (Alivernini et al., 2016). Several studies have shown that resveratrol can inhibit the expression of CD11B, CD14, and CD36 in PMA-induced macrophages. This finding suggests that resveratrol can inhibit the PMA-induced phosphorylation and nuclear translocation of PU.1, thus inhibiting monocyte differentiation into macrophages (Buhrmann et al., 2017).

**FIGURE 3:**
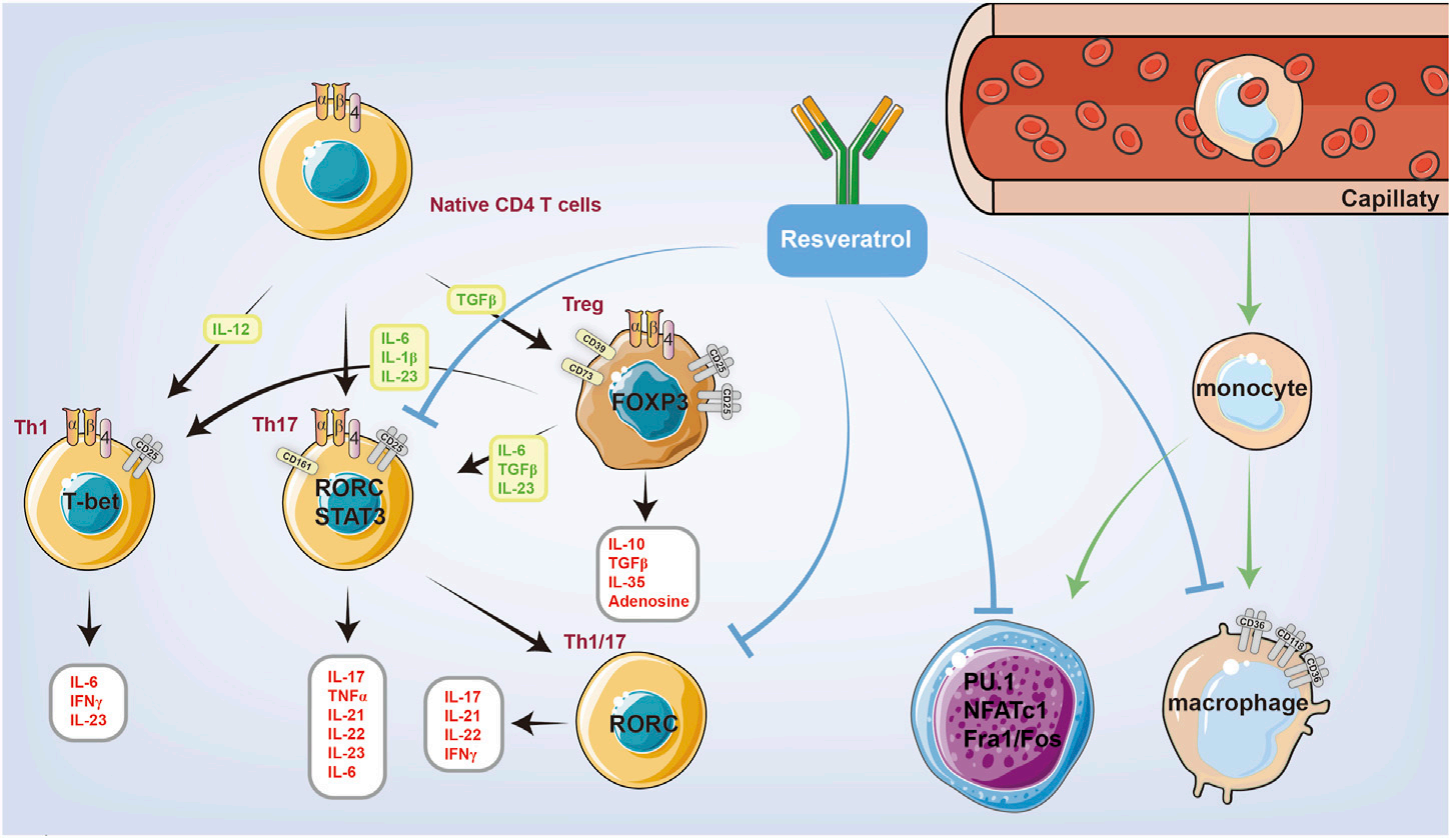
Resveratrol is closely related to innate immunity and autoimmunity, which can reduce the number of immune cells and inhibit the function of immune cells through intact mechanisms. Resveratrol can inhibit the PMA-induced phosphorylation and nuclear translocation of PU.1, thus inhibiting monocyte differentiation into macrophages. Resveratrol can also inhibit the function of Th1 and Th17 cells.

As an important part of the adaptive immune response, the activation of the T-cell system plays an important role in the pathological process of RA. In past research on RA, great attention has been paid to Th1; however, Th17 cells have attracted the interest of researchers due to their ability to produce a series of cytokines, such as IL17A, IL17F, IL21, IL22, and TNFα, that greatly promote RA development. Resveratrol can inhibit the TNF-β-mediated adhesion of T cells to chondrocytes as well as the production of CYP1A1 by inhibiting the AHR receptor, thus reducing the number and impairing the function of Th17 in DLNs (Figure 3).

### The effect of resveratrol on autophagy and mitochondrial energy metabolism

We believe that the decrease in autophagy and the disruption of mitochondrial energy metabolism may affect each other. Resveratrol can promote the production of mitochondrial reactive oxygen species (MTROS), decrease the levels of autophagic protein Beclin1, LC3A/B, and oxidative stress protein MnSOD, and promote FLS apoptosis by changing the mitochondrial membrane potential (Δψm)(Zhang et al., 2016). Another study showed that resveratrol can decrease the expression of autophagy-related proteins (LC3A/b, a, and TG-5). Downregulation of autophagy can lead to ROS accumulation. Subsequently, ROS can induce morphological changes in mitochondria that lead to mitochondrial dysfunction. Meanwhile, a decrease in the mitochondrial membrane potential leads to a decrease in intracellular ATP production, which results in a gradual decrease in intracellular calcium release and influx (Cao et al., 2018).

### Other resveratrol mechanisms remain to be thoroughly explored

Resveratrol has been shown to be an effective therapy for RA in several ways, and numerous mechanisms of action have been studied by hard-working scientists. However, owing to the complexity of its mechanism and the scope of its far-reaching impact, there remain many potential mechanisms worthy of further exploration. First, HIF-1α reaches abnormally high levels in RA FLS that may relate to angiogenesis. Several studies have shown that resveratrol inhibits the development of HIF-1α (Yang et al., 2018), and we hypothesized that this may also relate to the ability of resveratrol to inhibit RA. Furthermore, TNF-α plays an important role in RA pathogenesis, and recently, an entirely new concept has been proposed. Resveratrol inhibits the activation of the TNF-α-induced PI3/Akt signaling pathway in FLS, which inhibits the production of IL-1B and MMP3 (Tian et al., 2013; Gupta et al., 2014; Xu et al., 2017). Early research on resveratrol inhibition of the Wnt (Oz et al., 2019) and STAT3 pathways also provides new ideas, and the anti-inflammatory mechanism of resveratrol in these two pathways requires further investigation. Much of the literature we have read remains limited to measuring the expression of key target molecule mRNA throughout the joint, while the response of downstream immune components requires further investigation.

## Potential clinical applications of resveratrol in RA

The underlying mechanisms of resveratrol action are highly mature, and several clinical studies have been conducted (Mittal et al., 2021). In a recent study, resveratrol was shown to be effective in patients with RA. Specifically, the scientists divided the patients into two groups: a resveratrol-treated group and a control group. Interestingly, the differences in clinical and biochemical indices changed dramatically after the treatment period and favored the resveratrol-treated group. These clinical studies demonstrate that resveratrol plays an active role in RA. On the other hand, resveratrol was found to be effective as an adjuvant in a study of the effects of methotrexate alone and in combination with resveratrol (cell viability test). The results showed synergy between the two therapeutics (Lomholt et al., 2018). Compared with the traditional drug non-Norbert (in rats), resveratrol has been found to perform better (Wahba et al., 2016). In addition, resveratrol has been shown to have therapeutic potential for RA complications, such as atrial remodeling and atrial fibrillation (AF). Notably, resveratrol significantly reduced the rate and duration of AF in CIA rats and the elevation of IL-6 and TNF-α in the atria and serum of CIA mice, which reduced the occurrence of atrial apoptosis and fibrosis. In addition, resveratrol has been shown to reverse atrial energy metabolism dysfunction in CIA rats by inhibiting the AMPK/PGC1 α pathway (Zhang et al., 2018). Furthermore, resveratrol has been found to have protective effects on RA-induced atrial structure and metabolic remodeling and may provide a new therapeutic approach for RA-associated AF. Interstitial lung disease (ILD) is the most common pulmonary manifestation of RA, and resveratrol treatment was found to alleviate RA-ILD in rats by inhibiting the JAK/STAT/RANKL signaling pathway (Yang et al., 2019). With the occurrence of RA, patients are often susceptible to periodontitis. According to recent research, resveratrol may be effective in reducing periodontal disease by reducing the concentrations of tumor necrosis factors and interleukins (Chin et al., 2017; Javid et al., 2019). In conclusion, resveratrol has the potential to be used alone or as a supplement to improve treatment outcomes and prevent complications in patients with RA.

## Future expectations

The efficacy and mechanism of action of RSV in the treatment of RA have been extensively discussed. There is an urgent need to determine how to relate RSV to routine patient care. Here, we list several application methods for RSV, with the hope of catalyzing the development of new ideas and investigations.

### Drug delivery systems

**FIGURE 4:**
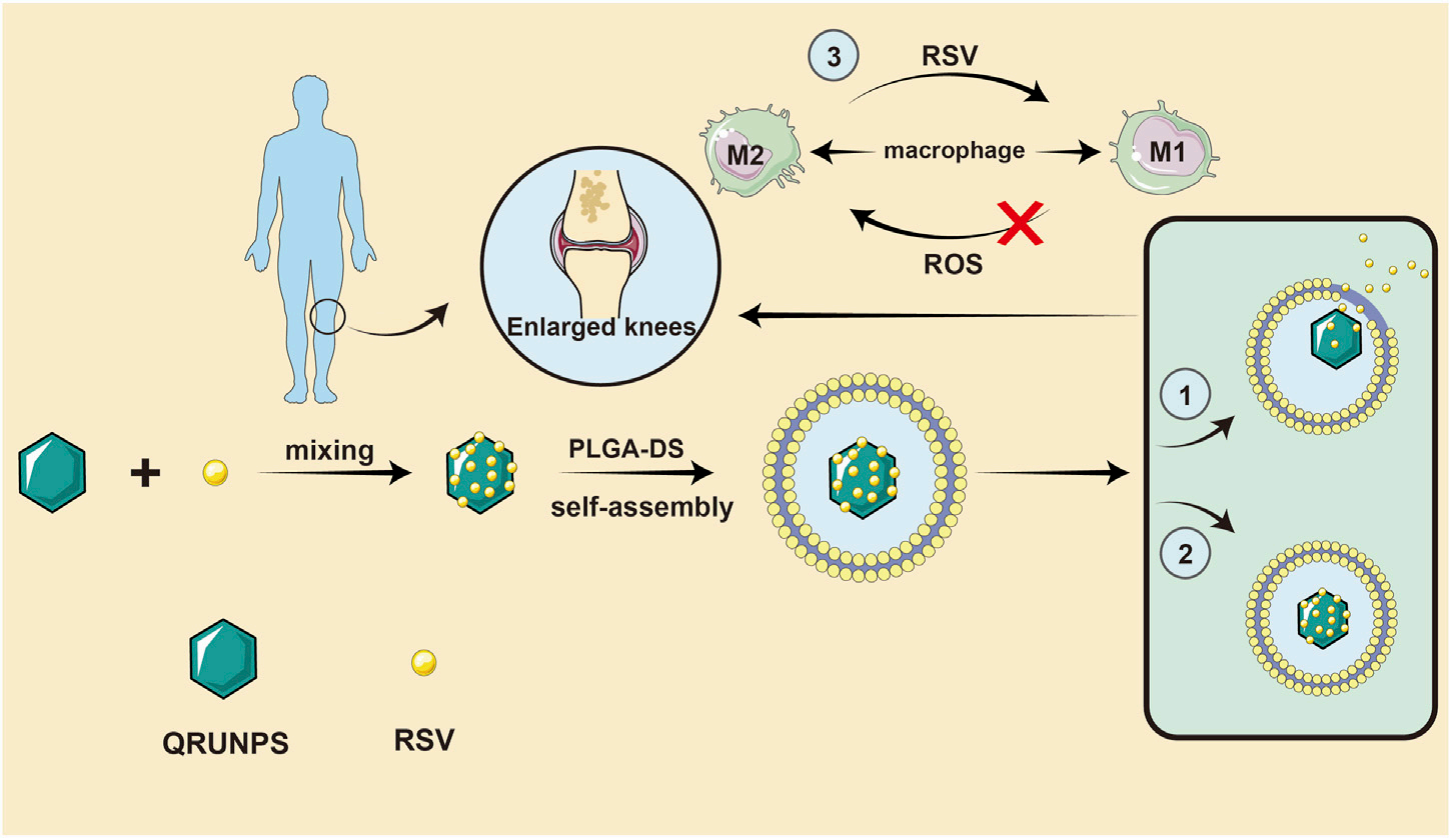
A drug delivery system idea for resveratrol. QRU-PLGA-RES-DS nanoparticles can effectively aggregate at the lesion site, promote the transformation of M2 macrophages, and reduce the recruitment of M1 macrophages, so as to effectively treat RA by eliminating an inflammatory response.

A drug delivery system (DDS) refers to a technical system for regulating the distribution of drugs in an organism with respect to space, time, and dose. Specifically, the goal is to deliver the right amount of drug to the right place at the right time, to increase the efficiency of drug use, improve efficacy, reduce costs, and reduce side effects. Chen et al. designed and synthesized a core-shell nanocomposite material (QRU-PLGA-RES-DS NPs). The core of the system was a tetragonal ruthenium nanoparticle (QRuNP) with a photothermal effect. The shell was composed of a thermosensitive molecular poly (lactic-glycolic acid) (PLGA) that was modified by the target molecule dextran sulfate (DS). The self-assembly of QRU-PLGA-DS nanoparticles effectively improved the water solubility and targeting properties of resveratrol (RES) (Chen et al., 2019). The QRU-PLGA-RES-DS nanoparticles significantly enhanced the ability of RES to reverse M1-TYPE macrophages to M2-TYPE macrophages by precision release. *In vivo* experiments further confirmed that under exogenous stimulation, QRU-PLGA-RES-DS nanoparticles can effectively aggregate at the lesion site, promote the transformation of M2 macrophages, and reduce the recruitment of M1 macrophages, so as to effectively treat RA by eliminating an inflammatory response. Thus, QRU-PLGA-RES-DS nanoparticles were found to effectively treat RA by reducing the inflammatory response. In addition, photoacoustic imaging (PA) of QRU nanoparticles can provide image guidance for the distribution and analysis of nanoparticles in inflammatory tissues. As RA is characterized by a long duration and recurrent attacks, it may be possible to achieve long-term controlled release of resveratrol by wrapping the compound in polymer layers. At the right temperature, the drug loading and release rates of QRU-PLGA-DS were significantly higher than those of liposomes, and investigators were able to control drug release rate by determining the appropriate temperature gradient. Notably, RA is a classic chronic disease that can be controlled by the long-term administration of drugs in the body, which DDS make possible. As the technology matures, the way resveratrol is administered will revolutionize. There is no doubt that with advances in drug delivery methods, resveratrol will compensate for the lack of other clinical advancements (Figure 4). In the future, we believe that resveratrol will be used in additional large-scale applications.

### Combination with radiation therapy

The administration of resveratrol during low-dose irradiation can significantly reduce mouse foot swelling and enhance the effect on serum TNFα, IL-1 β, inhibition of TBARs, and NOx levels. The relief of histopathological injury by dual treatment was found to be greater than that by single treatment. In addition, the expression of NF-κB (p65) in the synovial tissue under dual treatment was significantly inhibited, with similar efficacy to that of the diclofenac group. Therefore, the use of resveratrol in combination with radiotherapy is recommended.

### Current and future resveratrol investigation trends

Basic research on resveratrol is well established; however, large-scale multi-center clinical trials are lacking, and the exact effective dose and safety concerns (side effects) are yet to be established. Future research should focus on large-scale clinical studies and investigations into the pharmacology and other aspects of transformative medicinal research, with a dedication to finding sufficient medical evidence for the proper use of resveratrol in RA.

## Conclusion

Resveratrol, a natural phytohormone, has attracted much attention in recent years owing to its therapeutic effects on RA. It was found that resveratrol can inhibit mitogen-activated protein kinase (MAPK), nuclear factor κB (NF-κB), and other cellular signaling pathways, thereby inhibiting the production and release of interleukins, proinflammatory cytokines, and matrix metalloenzymes. In addition, resveratrol can induce the apoptosis of MH7A, FLS, and inflammatory cells in RA, regulate macrophage differentiation and function, and protect the normal redox kinetics of mitochondria. Furthermore, resveratrol can effectively relieve synovial inflammation, proliferation, and cartilage destruction in RA. In particular, resveratrol has been shown to reduce RA-related complications, such as periodontitis, AF, and interstitial pneumonia. Owing to the lack of sufficient clinical experimental evidence, resveratrol remains under investigation as a health care component. However, with the investigation of large patient samples, multi-center clinical research trials, and maturity and application of targeted, controlled-release, and other pharmaceutical technologies, resveratrol is expected to provide a new generation of anti-RA drugs.

## References

[B1] Alivernini S. Kurowska-Stolarska M. Tolusso B. Benvenuto R. Elmesmari A. Canestri S. (2016). MicroRNA-155 influences B-cell function through PU.1 in rheumatoid arthritis. Nat. Commun. 7, 12970. 10.1038/ncomms12970 27671860 PMC5052655

[B2] Alivernini S. MacDonald L. Elmesmari A. Finlay S. Tolusso B. Gigante M. R. (2020). Distinct synovial tissue macrophage subsets regulate inflammation and remission in rheumatoid arthritis. Nat. Med. 26 (8), 1295–1306. 10.1038/s41591-020-0939-8 32601335

[B3] Bartok B. Hammaker D. Firestein G. (2014). Phosphoinositide 3-kinase δ regulates migration and invasion of synoviocytes in rheumatoid arthritis. J. Immunol. 192 (5), 2063–2070. 10.4049/jimmunol.1300950 24470496

[B4] Bertolini D. R. Nedwin G. E. Bringman T. S. Smith D. D. Mundy G. R. (1986). Stimulation of bone resorption and inhibition of bone formation in vitro by human tumour necrosis factors. Nature 319 (6053), 516–518. 10.1038/319516a0 3511389

[B5] Bhoola K. Elson C. Dieppe P. (1992). Kinins--key mediators in inflammatory arthritis? Br. J. Rheumatol. 31 (8), 509–518. 10.1093/rheumatology/31.8.509 1643449

[B6] Biver E. Beague V. Verloop D. Mollet D. Lajugie D. Baudens G. (2009). Low and stable prevalence of rheumatoid arthritis in northern France. Jt. bone spine 76 (5), 497–500. 10.1016/j.jbspin.2009.03.013 19767228

[B7] Blanquer-Rosselló M. Hernández-López R. Roca P. Oliver J. Valle A. (2017). Resveratrol induces mitochondrial respiration and apoptosis in SW620 colon cancer cells. Biochim. Biophys. Acta. Gen. Subj. 1861 (2), 431–440. 10.1016/j.bbagen.2016.10.009 27760368

[B8] Breuss J. Atanasov A. Uhrin P. (2019). Resveratrol and its effects on the vascular system. Int. J. Mol. Sci. 20 (7), E1523. 10.3390/ijms20071523 30934670 PMC6479680

[B9] Buhrmann C. Popper B. Aggarwal B. Shakibaei M. (2017). Resveratrol downregulates inflammatory pathway activated by lymphotoxin α (TNF-β) in articular chondrocytes: Comparison with TNF-α. PloS one 12 (11), e0186993. 10.1371/journal.pone.0186993 29095837 PMC5667866

[B10] Byun H. Song J. Kim Y. Piao L. Won M. Park K. (2008a). Caspase-8 has an essential role in resveratrol-induced apoptosis of rheumatoid fibroblast-like synoviocytes. Rheumatol. Oxf. Engl. 47 (3), 301–308. 10.1093/rheumatology/kem368 18276737

[B11] Byun H. S. Song J. K. Kim Y. R. Piao L. Won M. Park K. A. (2008b). Caspase-8 has an essential role in resveratrol-induced apoptosis of rheumatoid fibroblast-like synoviocytes. Rheumatol. Oxf. 47 (3), 301–308. 10.1093/rheumatology/kem368 18276737

[B12] Cao W. Zhang J. Wang G. Lu J. Wang T. Chen X. (2018). Reducing-autophagy derived mitochondrial dysfunction during resveratrol promotes fibroblast-like synovial cell apoptosis. Anat. Rec. 301 (7), 1179–1188. 10.1002/ar.23798 29461680

[B13] Carbonell J. Cobo T. Balsa A. Descalzo M. Carmona L. (2008). The incidence of rheumatoid arthritis in Spain: Results from a nationwide primary care registry. Rheumatol. Oxf. Engl. 47 (7), 1088–1092. 10.1093/rheumatology/ken205 18511475

[B14] Carter L. D'Orazio J. Pearson K. (2014). Resveratrol and cancer: Focus on in vivo evidence. Endocr. Relat. Cancer 21 (3), R209–R225. 10.1530/erc-13-0171 24500760 PMC4013237

[B15] Chan S. Kantham S. Rao V. Palanivelu M. Pham H. Shaw P. (2016). Metal chelation, radical scavenging and inhibition of Aβ₄₂ fibrillation by food constituents in relation to Alzheimer's disease. Food Chem. 199, 185–194. 10.1016/j.foodchem.2015.11.118 26775960

[B16] Chen X. Zhu X. Ma L. Lin A. Gong Y. Yuan G. (2019). A core-shell structure QRu-PLGA-RES-DS NP nanocomposite with photothermal response-induced M2 macrophage polarization for rheumatoid arthritis therapy. Nanoscale 11 (39), 18209–18223. 10.1039/c9nr05922a 31560010

[B17] Chin Y. Cheng G. Shih Y. Lin C. Lin S. Lai H. (2017). Therapeutic applications of resveratrol and its derivatives on periodontitis. Ann. N. Y. Acad. Sci. 1403 (1), 101–108. 10.1111/nyas.13433 28856691

[B18] Cock I. van Vuuren S. (2014). Anti-Proteus activity of some South African medicinal plants: Their potential for the prevention of rheumatoid arthritis. Inflammopharmacology 22 (1), 23–36. 10.1007/s10787-013-0179-3 23877712

[B19] Cock I. Winnett V. Sirdaarta J. Matthews B. (2015). The potential of selected Australian medicinal plants with anti-Proteus activity for the treatment and prevention of rheumatoid arthritis. Pharmacogn. Mag. 11, S190–S208. 10.4103/0973-1296.157734 26109767 PMC4461961

[B20] Crofford L. (1997). COX-1 and COX-2 tissue expression: Implications and predictions. J. Rheumatol. Suppl. 49, 15–19. 9249646

[B21] Crowson C. Matteson E. Myasoedova E. Michet C. Ernste F. Warrington K. (2011). The lifetime risk of adult-onset rheumatoid arthritis and other inflammatory autoimmune rheumatic diseases. Arthritis Rheum. 63 (3), 633–639. 10.1002/art.30155 21360492 PMC3078757

[B22] de Oliveira M. Nabavi S. Manayi A. Daglia M. Hajheydari Z. Nabavi S. (2016). Resveratrol and the mitochondria: From triggering the intrinsic apoptotic pathway to inducing mitochondrial biogenesis, a mechanistic view. Biochim. Biophys. Acta 1860 (4), 727–745. 10.1016/j.bbagen.2016.01.017 26802309

[B23] Dedmon L. E. (2020). The genetics of rheumatoid arthritis. Rheumatol. Oxf. 59 (10), 2661–2670. 10.1093/rheumatology/keaa232 32638005

[B24] Doran M. Pond G. Crowson C. O'Fallon W. Gabriel S. (2002). Trends in incidence and mortality in rheumatoid arthritis in Rochester, Minnesota, over a forty-year period. Arthritis Rheum. 46 (3), 625–631. 10.1002/art.509 11920397

[B25] Dudics S. Langan D. Meka R. R. Venkatesha S. H. Berman B. M. Che C. T. (2018b). Natural products for the treatment of autoimmune arthritis: Their mechanisms of action, targeted delivery, and interplay with the host microbiome. Int. J. Mol. Sci. 19 (9), E2508. 10.3390/ijms19092508 30149545 PMC6164747

[B26] Dudics S. Langan D. Meka R. Venkatesha S. Berman B. Che C. (2018a). Natural products for the treatment of autoimmune arthritis: Their mechanisms of action, targeted delivery, and interplay with the host microbiome. Int. J. Mol. Sci. 19 (9), E2508. 10.3390/ijms19092508 30149545 PMC6164747

[B27] el-Dahr S. Dipp S. Yosipiv I. Baricos W. (1996). Bradykinin stimulates c-fos expression, AP-1-DNA binding activity and proliferation of rat glomerular mesangial cells. Kidney Int. 50 (6), 1850–1855. 10.1038/ki.1996.505 8943466

[B28] Elmali N. Baysal O. Harma A. Esenkaya I. Mizrak B. (2007). Effects of resveratrol in inflammatory arthritis. Inflammation 30, 1–6. 10.1007/s10753-006-9012-0 17115116

[B29] Elmali N. Esenkaya I. Harma A. Ertem K. Turkoz Y. Mizrak B. (2005). Effect of resveratrol in experimental osteoarthritis in rabbits. Inflamm. Res. 54 (4), 158–162. 10.1007/s00011-004-1341-6 15883738

[B30] Fan W. Zhou Z. Huang X. Bao C. Du F. (2013). Deoxycytidine kinase promotes the migration and invasion of fibroblast-like synoviocytes from rheumatoid arthritis patients. Int. J. Clin. Exp. Pathol. 6 (12), 2733–2744. 24294360 PMC3843254

[B31] Gao J. Zheng W. Wang L. Song B. (2015). A disintegrin and metallproteinase 15 knockout decreases migration of fibroblast-like synoviocytes and inflammation in rheumatoid arthritis. Mol. Med. Rep. 11 (6), 4389–4396. 10.3892/mmr.2015.3302 25650586

[B32] Gavrilă B. Ciofu C. Stoica V. (2016). Biomarkers in rheumatoid arthritis, what is new? J. Med. Life 9 (2), 144–148. 27453744 PMC4863504

[B33] Gibellini L. Bianchini E. De Biasi S. Nasi M. Cossarizza A. Pinti M. (2015). Natural compounds modulating mitochondrial functions. Evid. Based. Complement. Altern. Med. 2015, 527209. 10.1155/2015/527209 PMC4489008 26167193

[B34] Glehr M. Fritsch-Breisach M. Lohberger B. Walzer S. M. Moazedi-Fuerst F. Rinner B. (2013b). Influence of resveratrol on rheumatoid fibroblast-like synoviocytes analysed with gene chip transcription. Phytomedicine 20 (3-4), 310–318. 10.1016/j.phymed.2012.09.020 23137833

[B35] Glehr M. Fritsch-Breisach M. Lohberger B. Walzer S. Moazedi-Fuerst F. Rinner B. (2013a). Influence of resveratrol on rheumatoid fibroblast-like synoviocytes analysed with gene chip transcription. Phytomedicine 20, 310–318. 10.1016/j.phymed.2012.09.020 23137833

[B36] Grell M. Douni E. Wajant H. Lohden M. Clauss M. Maxeiner B. (1995). The transmembrane form of tumor necrosis factor is the prime activating ligand of the 80 kDa tumor necrosis factor receptor. Cell. 83 (5), 793–802. 10.1016/0092-8674(95)90192-2 8521496

[B37] Gupta S. Tyagi A. Deshmukh-Taskar P. Hinojosa M. Prasad S. Aggarwal B. (2014). Downregulation of tumor necrosis factor and other proinflammatory biomarkers by polyphenols. Arch. Biochem. Biophys. 559, 91–99. 10.1016/j.abb.2014.06.006 24946050

[B38] Hao L. Wan Y. Xiao J. Tang Q. Deng H. Chen L. (2017). A study of Sirt1 regulation and the effect of resveratrol on synoviocyte invasion and associated joint destruction in rheumatoid arthritis. Mol. Med. Rep. 16 (4), 5099–5106. 10.3892/mmr.2017.7299 28849139 PMC5647035

[B39] Harris E. (1990). Rheumatoid arthritis. Pathophysiology and implications for therapy. N. Engl. J. Med. 322 (18), 1277–1289. 10.1056/nejm199005033221805 2271017

[B40] Hasan M. Bae H. (2017). An overview of stress-induced resveratrol synthesis in grapes: Perspectives for resveratrol-enriched grape products. Molecules 22 (2), E294. 10.3390/molecules22020294 28216605 PMC6155908

[B41] Hsieh T. Wu J. (2010). Resveratrol: Biological and pharmaceutical properties as anticancer molecule. BioFactors Oxf. Engl. 36 (5), 360–369. 10.1002/biof.105 PMC3655417 20623546

[B42] Jardim F. de Rossi F. Nascimento M. da Silva Barros R. Borges P. Prescilio I. (2018). Resveratrol and brain mitochondria: A review. Mol. Neurobiol. 55 (3), 2085–2101. 10.1007/s12035-017-0448-z 28283884

[B43] Javid A. Hormoznejad R. Yousefimanesh H. Haghighi-Zadeh M. Zakerkish M. (2019). Impact of resveratrol supplementation on inflammatory, antioxidant, and periodontal markers in type 2 diabetic patients with chronic periodontitis. Diabetes Metab. Syndr. 13 (4), 2769–2774. 10.1016/j.dsx.2019.07.042 31405706

[B44] Jie L. Huang R. Sun W. Wei S. Chu Y. Huang Q. (2015). Role of cysteine-rich angiogenic inducer 61 in fibroblast-like synovial cell proliferation and invasion in rheumatoid arthritis. Mol. Med. Rep. 11 (2), 917–923. 10.3892/mmr.2014.2770 25351421 PMC4262486

[B45] Kaipiainen-Seppanen O. Kautiainen H. (2006). Declining trend in the incidence of rheumatoid factor-positive rheumatoid arthritis in Finland 1980-2000. J. Rheumatol. 33 (11), 2132–2138. 17014003

[B46] Kalantari H. Das D. (2010). Physiological effects of resveratrol. BioFactors Oxf. Engl. 36 (5), 401–406. 10.1002/biof.100 20623511

[B47] Kauppinen A. Suuronen T. Ojala J. Kaarniranta K. Salminen A. (2013). Antagonistic crosstalk between NF-κB and SIRT1 in the regulation of inflammation and metabolic disorders. Cell. Signal. 25 (10), 1939–1948. 10.1016/j.cellsig.2013.06.007 23770291

[B48] Khojah H. Ahmed S. Abdel-Rahman M. Elhakeim E. (2018). Resveratrol as an effective adjuvant therapy in the management of rheumatoid arthritis: A clinical study. Clin. Rheumatol. 37 (8), 2035–2042. 10.1007/s10067-018-4080-8 29611086

[B49] Klareskog L. van der Heijde D. de Jager J. Gough A. Kalden J. Malaise M. (2004). Therapeutic effect of the combination of etanercept and methotrexate compared with each treatment alone in patients with rheumatoid arthritis: Double-blind randomised controlled trial. Lancet (London, Engl. 363 (9410), 675–681. 10.1016/s0140-6736(04)15640-7 15001324

[B50] Li D. Xiao Z. Wang G. Song X. (2015). Knockdown of ADAM10 inhibits migration and invasion of fibroblast-like synoviocytes in rheumatoid arthritis. Mol. Med. Rep. 12 (4), 5517–5523. 10.3892/mmr.2015.4011 26135838

[B51] Lomholt S. Mellemkjaer A. Iversen M. Pedersen S. Kragstrup T. (2018). Resveratrol displays anti-inflammatory properties in an *ex vivo* model of immune mediated inflammatory arthritis. BMC Rheumatol. 2, 27. 10.1186/s41927-018-0036-5 30886977 PMC6390607

[B52] Marahleh A. Kitaura H. Ohori F. Kishikawa A. Ogawa S. Shen W. R. (2019). TNF-Alpha directly enhances osteocyte RANKL expression and promotes osteoclast formation. Front. Immunol. 10, 2925. 10.3389/fimmu.2019.02925 31921183 PMC6923682

[B53] Marrelli A. Cipriani P. Liakouli V. Carubbi F. Perricone C. Perricone R. (2011). Angiogenesis in rheumatoid arthritis: A disease specific process or a common response to chronic inflammation? Autoimmun. Rev. 10 (10), 595–598. 10.1016/j.autrev.2011.04.020 21545851

[B54] McInnes I. Schett G. (2011). The pathogenesis of rheumatoid arthritis. N. Engl. J. Med. 365 (23), 2205–2219. 10.1056/NEJMra1004965 22150039

[B55] Micheau O. Tschopp J. (2003). Induction of TNF receptor I-mediated apoptosis via two sequential signaling complexes. Cell. 114 (2), 181–190. 10.1016/s0092-8674(03)00521-x 12887920

[B56] Miller M. Manning H. Jain A. Troeberg L. Dudhia J. Essex D. (2009). Membrane type 1 matrix metalloproteinase is a crucial promoter of synovial invasion in human rheumatoid arthritis. Arthritis Rheum. 60 (3), 686–697. 10.1002/art.24331 19248098 PMC2819053

[B57] Mittal M. Mehta P. Rajput S. Rajender S. Chattopadhyay N. (2021). The pharmacological assessment of resveratrol on preclinical models of rheumatoid arthritis through a systematic review and meta-analysis. Eur. J. Pharmacol. 910, 174504. 10.1016/j.ejphar.2021.174504 34520733

[B58] Nakayama H. Yaguchi T. Yoshiya S. Nishizaki T. (2012a). Resveratrol induces apoptosis MH7A human rheumatoid arthritis synovial cells in a sirtuin 1-dependent manner. Rheumatol. Int. 32 (1), 151–157. 10.1007/s00296-010-1598-8 20697895 PMC3253293

[B59] Nakayama H. Yaguchi T. Yoshiya S. Nishizaki T. (2012b). Resveratrol induces apoptosis MH7A human rheumatoid arthritis synovial cells in a sirtuin 1-dependent manner. Rheumatol. Int. 32 (1), 151–157. 10.1007/s00296-010-1598-8 20697895 PMC3253293

[B60] Nguyen N. Nakahama T. Nguyen C. Tran T. Le V. Chu H. (2015a). Aryl hydrocarbon receptor antagonism and its role in rheumatoid arthritis. J. Exp. Pharmacol. 7, 29–35. 10.2147/jep.S63549 27186143 PMC4863532

[B61] Nguyen N. T. Nakahama T. Nguyen C. H. Tran T. T. Le V. S. Chu H. H. (2015b). Aryl hydrocarbon receptor antagonism and its role in rheumatoid arthritis. J. Exp. Pharmacol. 7, 29–35. 10.2147/jep.S63549 27186143 PMC4863532

[B62] Nunes S. Danesi F. Del Rio D. Silva P. (2018). Resveratrol and inflammatory bowel disease: The evidence so far. Nutr. Res. Rev. 31 (1), 85–97. 10.1017/s095442241700021x 29191255

[B63] Oz B. Yildirim A. Yolbas S. Celik Z. Etem E. Deniz G. (2019). Resveratrol inhibits Src tyrosine kinase, STAT3, and Wnt signaling pathway in collagen induced arthritis model. BioFactors Oxf. Engl. 45 (1), 69–74. 10.1002/biof.1463 30496633

[B64] Park S. Lee S. Baek S. Lee C. Lee W. Rhim B. (2013). Suppression of PU.1-linked TLR4 expression by cilostazol with decrease of cytokine production in macrophages from patients with rheumatoid arthritis. Br. J. Pharmacol. 168 (6), 1401–1411. 10.1111/bph.12021 23072581 PMC3596645

[B65] Park S. Lee S. Kim H. Lee S. Lee W. Hong K. (2016). SIRT1 inhibits differentiation of monocytes to macrophages: Amelioration of synovial inflammation in rheumatoid arthritis. J. Mol. Med. 94 (8), 921–931. 10.1007/s00109-016-1402-7 26956118

[B66] Pedersen J. Kjaer N. Svendsen A. Hørslev-Petersen K. (2009). Incidence of rheumatoid arthritis from 1995 to 2001: Impact of ascertainment from multiple sources. Rheumatol. Int. 29 (4), 411–415. 10.1007/s00296-008-0713-6 18853167

[B67] Price N. L. Gomes A. P. Ling A. J. Duarte F. V. Martin-Montalvo A. North B. J. (2012). SIRT1 is required for AMPK activation and the beneficial effects of resveratrol on mitochondrial function. Cell. Metab. 15 (5), 675–690. 10.1016/j.cmet.2012.04.003 22560220 PMC3545644

[B68] Sano H. Hla T. Maier J. Crofford L. Case J. Maciag T. (1992). In vivo cyclooxygenase expression in synovial tissues of patients with rheumatoid arthritis and osteoarthritis and rats with adjuvant and streptococcal cell wall arthritis. J. Clin. Invest. 89 (1), 97–108. 10.1172/jci115591 1729286 PMC442824

[B69] Scott D. Wolfe F. Huizinga T. (2010). Rheumatoid arthritis. Lancet (London, Engl. 376 (9746), 1094–1108. 10.1016/s0140-6736(10)60826-4 20870100

[B70] Shakibaei M. Harikumar K. Aggarwal B. (2009). Resveratrol addiction: To die or not to die. Mol. Nutr. Food Res. 53 (1), 115–128. 10.1002/mnfr.200800148 19072742

[B71] Shin J. I. Lee K. H. Joo Y. H. Lee J. M. Jeon J. Jung H. J. (2019). Inflammasomes and autoimmune and rheumatic diseases: A comprehensive review. J. Autoimmun. 103, 102299. 10.1016/j.jaut.2019.06.010 31326231

[B72] Singh J. Saag K. Bridges S. Akl E. Bannuru R. Sullivan M. (2016). 2015 American college of rheumatology guideline for the treatment of rheumatoid arthritis. Arthritis Care Res. 68 (1), 1–25. 10.1002/acr.22783 26545825

[B73] Sparks J. A. (2019). Rheumatoid arthritis. Ann. Intern. Med. 170 (1)–ITC16. 10.7326/aitc201901010 30596879

[B74] Symmons D. Turner G. Webb R. Asten P. Barrett E. Lunt M. (2002). The prevalence of rheumatoid arthritis in the United Kingdom: New estimates for a new century. Rheumatol. Oxf. Engl. 41 (7), 793–800. 10.1093/rheumatology/41.7.793 12096230

[B75] Szapłyko W. Gromek I. Józwiak S. Karkowska B. Skobejko-Włodarska L. (2003). Evaluation of the central nervous system in children with bladder dysfunction with the use of evoked potentials. Przegl. Lek. 60 Suppl 1, 45–47. 12945162

[B76] Tellone E. Galtieri A. Russo A. Giardina B. Ficarra S. (2015). Resveratrol: A focus on several neurodegenerative diseases. Oxid. Med. Cell. Longev. 2015, 392169. 10.1155/2015/392169 26180587 PMC4477222

[B77] Tian J. Chen J. Gao J. Li L. Xie X. (2013). Resveratrol inhibits TNF-α-induced IL-1β, MMP-3 production in human rheumatoid arthritis fibroblast-like synoviocytes via modulation of PI3kinase/Akt pathway. Rheumatol. Int. 33 (7), 1829–1835. 10.1007/s00296-012-2657-0 23328930

[B78] Timmers S. Konings E. Bilet L. Houtkooper R. H. van de Weijer T. Goossens G. H. (2011). Calorie restriction-like effects of 30 days of resveratrol supplementation on energy metabolism and metabolic profile in obese humans. Cell. Metab. 14 (5), 612–622. 10.1016/j.cmet.2011.10.002 22055504 PMC3880862

[B79] Tsai M. Hsu L. Lee C. Chiang Y. Lee M. How J. (2017). Resveratrol inhibits urban particulate matter-induced COX-2/PGE2 release in human fibroblast-like synoviocytes via the inhibition of activation of NADPH oxidase/ROS/NF-κB. Int. J. Biochem. Cell. Biol. 88, 113–123. 10.1016/j.biocel.2017.05.015 28495310

[B80] Valero T. (2014). Mitochondrial biogenesis: Pharmacological approaches. Curr. Pharm. Des. 20 (35), 5507–5509. 10.2174/138161282035140911142118 24606795

[B81] van der Helm-van Mil A. H. Huizinga T. W. Schreuder G. M. Breedveld F. C. de Vries R. R. Toes R. E. (2005). An independent role of protective HLA class II alleles in rheumatoid arthritis severity and susceptibility. Arthritis Rheum. 52 (9), 2637–2644. 10.1002/art.21272 16142711

[B82] Wahba M. Messiha B. Abo-Saif A. (2016). Protective effects of fenofibrate and resveratrol in an aggressive model of rheumatoid arthritis in rats. Pharm. Biol. 54 (9), 1705–1715. 10.3109/13880209.2015.1125931 26704826

[B83] Wang G. Xie X. Yuan L. Qiu J. Duan W. Xu B. (2020). Resveratrol ameliorates rheumatoid arthritis via activation of SIRT1-Nrf2 signaling pathway. BioFactors Oxf. Engl. 46 (3), 441–453. 10.1002/biof.1599 31883358

[B84] Wang H. Wang H. Huang S. Zhao H. Cao Y. Wang G. (2014). Inhibitory effect of baicalin on collagen-induced arthritis in rats through the nuclear factor-κB pathway. J. Pharmacol. Exp. Ther. 350 (2), 435–443. 10.1124/jpet.114.215145 24893986

[B85] Wasserman A. (2011). Diagnosis and management of rheumatoid arthritis. Am. Fam. Physician 84 (11), 1245–1252. 22150658

[B86] Wendling D. Abbas W. Godfrin-Valnet M. Kumar A. Guillot X. Khan K. (2015). Dysregulated serum IL-23 and SIRT1 activity in peripheral blood mononuclear cells of patients with rheumatoid arthritis. PloS one 10 (3), e0119981. 10.1371/journal.pone.0119981 25799392 PMC4370395

[B87] Wendling D. Vidon C. Abbas W. Guillot X. Toussirot E. Herbein G. (2014). Sirt1 activity in peripheral blood mononuclear cells from patients with rheumatoid arthritis. Jt. bone spine 81 (5), 462–463. 10.1016/j.jbspin.2014.02.006 24746477

[B88] Xia N. Daiber A. Förstermann U. Li H. (2017). Antioxidant effects of resveratrol in the cardiovascular system. Br. J. Pharmacol. 174 (12), 1633–1646. 10.1111/bph.13492 27058985 PMC5446570

[B89] Xie P. Browning D. Hay N. Mackman N. Ye R. (2000). Activation of NF-kappa B by bradykinin through a Galpha(q)- and Gbeta gamma-dependent pathway that involves phosphoinositide 3-kinase and Akt. J. Biol. Chem. 275 (32), 24907–24914. 10.1074/jbc.M001051200 10801799

[B90] Xu B. Wang G. Zhang J. Cao W. Chen X. (2017). Resveratrol decreases FoXO protein expression through PI3K-Akt-dependent pathway inhibition in H₂O₂-treated synoviocytes. Histol. Histopathol. 32 (12), 1305–1315. 10.14670/hh-11-884 28211035

[B91] Xuzhu G. Komai-Koma M. Leung B. Howe H. McSharry C. McInnes I. (2012). Resveratrol modulates murine collagen-induced arthritis by inhibiting Th17 and B-cell function. Ann. Rheum. Dis. 71 (1), 129–135. 10.1136/ard.2011.149831 21953348

[B92] Yang C. Chen Y. Chi P. Lin C. Hsiao L. (2017). Resveratrol inhibits BK-induced COX-2 transcription by suppressing acetylation of AP-1 and NF-κB in human rheumatoid arthritis synovial fibroblasts. Biochem. Pharmacol. 132, 77–91. 10.1016/j.bcp.2017.03.003 28288820

[B93] Yang G. Chang C. Yang Y. Yuan L. Xu L. Ho C. (2018). Resveratrol alleviates rheumatoid arthritis via reducing ROS and inflammation, inhibiting MAPK signaling pathways, and suppressing angiogenesis. J. Agric. Food Chem. 66 (49), 12953–12960. 10.1021/acs.jafc.8b05047 30511573

[B94] Yang G. Lyu L. Wang X. Bao L. Lyu B. Lin Z. (2019). Systemic treatment with resveratrol alleviates adjuvant arthritis-interstitial lung disease in rats via modulation of JAK/STAT/RANKL signaling pathway. Pulm. Pharmacol. Ther. 56, 69–74. 10.1016/j.pupt.2019.03.011 30930172

[B95] Yang X. Karsenty G. (2002). Transcription factors in bone: Developmental and pathological aspects. Trends Mol. Med. 8 (7), 340–345. 10.1016/s1471-4914(02)02340-7 12114114

[B96] Yanni G. Whelan A. Feighery C. Bresnihan B. (1994). Synovial tissue macrophages and joint erosion in rheumatoid arthritis. Ann. Rheum. Dis. 53 (1), 39–44. 10.1136/ard.53.1.39 8311554 PMC1005241

[B97] Yeung F. Hoberg J. Ramsey C. Keller M. Jones D. Frye R. (2004). Modulation of NF-kappaB-dependent transcription and cell survival by the SIRT1 deacetylase. EMBO J. 23 (12), 2369–2380. 10.1038/sj.emboj.7600244 15152190 PMC423286

[B98] Zhang J. Song X. Cao W. Lu J. Wang X. Wang G. (2016). Autophagy and mitochondrial dysfunction in adjuvant-arthritis rats treatment with resveratrol. Sci. Rep. 6, 32928. 10.1038/srep32928 27611176 PMC5017199

[B99] Zhang Y. Wang G. Wang T. Cao W. Zhang L. Chen X. (2019). Nrf2-Keap1 pathway-mediated effects of resveratrol on oxidative stress and apoptosis in hydrogen peroxide-treated rheumatoid arthritis fibroblast-like synoviocytes. Ann. N. Y. Acad. Sci. 1457 (1), 166–178. 10.1111/nyas.14196 31475364

[B100] Zhang Y. Zhang S. Liu Z. Zhao X. Yuan Y. Sheng L. (2018). Resveratrol prevents atrial fibrillation by inhibiting atrial structural and metabolic remodeling in collagen-induced arthritis rats. Naunyn. Schmiedeb. Arch. Pharmacol. 391 (11), 1179–1190. 10.1007/s00210-018-1554-9 30135998

